# Borderline Ovarian Tumors and Diagnostic Dilemma of Intraoperative Diagnosis: Could Preoperative He4 Assay and ROMA Score Assessment Increase the Frozen Section Accuracy? A Multicenter Case-Control Study

**DOI:** 10.1155/2014/803598

**Published:** 2014-11-05

**Authors:** Salvatore Gizzo, Roberto Berretta, Stefania Di Gangi, Maria Guido, Giuliano Carlo Zanni, Ilaria Franceschetti, Michela Quaranta, Mario Plebani, Giovanni Battista Nardelli, Tito Silvio Patrelli

**Affiliations:** ^1^Department of Woman and Child Health, University of Padua, 35128 Padua, Italy; ^2^Dipartimento di Salute della Donna e del Bambino, U.O.C. di Ginecologia e Ostetricia, Via Giustiniani 3, 35128 Padova, Italy; ^3^Department of Surgical Sciences, University of Parma, 43100 Parma, Italy; ^4^Department of Medical Diagnostic and Special Therapy, University of Padua, 35128 Padua, Italy; ^5^Gynaecologic and Obstetric Units, Vicenza General Hospital, 36100 Vicenza, Italy; ^6^Department of Obstetrics and Gynaecology, University of Verona, 37100 Verona, Italy; ^7^Department of Laboratory Medicine, University of Padua, 35128 Padua, Italy

## Abstract

The aim of our study was to assess the value of a preoperative He4-serum-assay and ROMA-score assessment in improving the accuracy of frozen section histology in the diagnosis of borderline ovarian tumors (BOT). 113 women presenting with a unilateral ovarian mass diagnosed as serous/mucinous BOT at frozen-section-histology (FS) and/or confirmed on final pathology were recruited. Pathologists were informed of the results of preoperative clinical/instrumental assessment of all patients. For Group_A patients, additional information regarding He4, CA125, and ROMA score was available (in Group_B only CA125 was known). The comparison between Group A and Group B in terms of FS accuracy, demonstrated a consensual diagnosis in 62.8% versus 58.6% (*P*: n.s.), underdiagnosis in 25.6% versus 41.4% (*P* < 0.05), and overdiagnosis in 11.6% versus 0% (*P* < 0.01). Low FS diagnostic accuracy was associated with menopausal status (OR: 2.13), laparoscopic approach (OR: 2.18), mucinous histotype (OR: 2.23), low grading (OR: 1.30), and FIGO stage I (OR: 2.53). Ultrasound detection of papillae (OR: 0.29), septa (OR: 0.39), atypical vascularization (OR: 0.34), serum He4 assay (OR: 0.39), and ROMA score assessment (OR: 0.44) decreased the probability of underdiagnosis. A combined preoperative assessment through serum markers and ultrasonographic features may potentially reduce the risk of underdiagnosis of BOTs on FS while likely increasing the concomitant incidence of false-positive events.

## 1. Introduction

Borderline ovarian tumors (BOTs) are an independent identity which represents a unique intermediate stage of ovarian neoplasia. Although certain histological features suggest malignant behavior, they are classified as tumors of “low malignant potential” since their prognosis is excellent when compared with that of invasive ovarian carcinoma. Histologically they are characterized by the absence of stromal invasion despite, in distinction from benign forms, demonstrating an increased mitotic index and the nuclear atypia [1].

BOTs account for approximately 10–15% of ovarian epithelial tumors; however, the true incidence of the disease remains unknown. BOTs can be unilateral or bilateral. Typically, BOTs remain confined to one or both ovaries but rarely may spread to the peritoneum and lymph nodes. Even in the unlikely event of tumor spread, the prognosis is generally favorable with a 10-year survival rate higher than 95%. Nevertheless cases of recurrence and disease-related deaths have been reported [2]. The majority of these tumors are diagnosed in the 20- and 46-year-old age group with about 25% of the cases occurring in women younger than 35 years and likely having the desire to preserve child-bearing potential.

This fact has generated many debates concerning the extent of surgical treatment representing standard of care. Currently consensus regarding the identification of those cases which may benefit from fertility sparing surgery rather than a radical approach has not been yet achieved.

The oncologic dilemma is based on evidence that conservative fertility surgery is associated with an increased rate of recurrence (35% of the cases) compared to the 5% observed in patients treated with radical surgery [3].

Preoperative assessment is based on imaging techniques (usually transvaginal ultrasound—TVS) and biochemical serum markers assays (CA125 and He4) [4, 5].

Ultrasound can give information concerning morphology such as cystic echo pattern, septa presence, endocystic vegetation, and atypical vascularization patterns, yet it often fails to confirm diagnostic suspicions even when applying the “pattern recognition” criteria to differentiate benign and malignant adnexal masses [4]. CA125 is the most widely used marker and correlates closely with ovarian malignancy. Its specificity, however, is far from ideal [6]. The introduction of human epididymis 4 (HE4) marker and risk of ovarian malignancy algorithm (ROMA) into clinical practice has improved the specificity of CA125 for the detection of ovarian malignancy and increased our capability to differentiate benign from malignant ovarian masses [5].

A preoperative definite diagnosis of an atypical adnexal mass is currently impossible to achieve with reasonable accuracy; thus intraoperative histology remains pivotal in defining the surgical treatment of choice [7]. While frozen section has a high overall accuracy for the diagnosis of ovarian malignancy, it is of limited validity in intraoperative identification of BOTs (high rate of false-negative and false-positive diagnosis).

This often induces surgeons to postpone the appropriate surgical staging until after definitive pathology, thus potentially increasing the rate of reintervention, postsurgical tumor spread, delay in adjuvant treatment, and increased psychological distress especially in young women with high fertility desire [8–13].

The implementation of FS for the diagnosis of ovarian masses has been studied extensively, reaching a positive predictive value of over 90% for benign and malignant lesions [8]. However, the diagnostic accuracy of FS in BOTs has been less well characterized with reported rates ranging from 56 to 89% [9–12]. Since BOTs are relatively rare, information available in literature is often poor and fragmentary; thus, the clinical-pathological factors influencing the likelihood accuracy of BOTs FS are not well understood.

Recently, several attempts to define both sonographic and histological features able to increase the FS diagnostic accuracy in BOTs have been made, yet the role of preoperative serum markers assays in diagnosis remains uninvestigated [9–12]. CA125 serum level is the only proposed and investigated marker, but it seems unable to improve the accuracy of FS diagnosis [9]. He4 serum marker increases CA125 specificity in preoperative detection of ovarian malignancy both alone and in combination with CA125 (ROMA score). To our knowledge, only one study investigated He4 ovarian expression profiles in BOTs, healthy and malignant ovarian tissue [14].

We conducted a multicenter study to assess the value of a preoperative assessment by He4 serum assay and ROMA score in improving the accuracy of frozen section histology in the diagnosis of borderline ovarian tumors in pre- and postmenopausal women.

## 2. Methods

We conducted a retrospective multicenter case-control study on women with unilateral pelvic ovarian mass referred for adequate surgical treatment, in accordance with FIGO guidelines, between March 2010 and September 2013 to the following centres: University of Padua (Gynecological Clinic-Women and Child Health Department), University of Parma (Gynecological Unit-Surgical Sciences Department), and Vicenza General Hospital (Gyn/Ob Unit) [7].

We focused on a cohort of patients who underwent intraoperative FS histologic analysis of adnexal masses with a subsequent diagnosis of serous/mucinous BOTs either at FS and/or at definitive pathology (postoperative).

As inclusion criteria, we considered as mandatory the availability of data regarding the following: patient characteristics (age, parity, BMI, and hormonal status), preoperative sonographic features of the ovarian mass (size, septa, atypical vascularization, echogenicity, and papillary patterns), preoperative CA125 serum level, surgical approach chosen (laparotomy or laparoscopy, cystectomy, or salpingo-oophorectomy), FS and final pathology (histotype, grading, and FIGO stage), and peritoneal cytology.

All FS diagnoses were performed by experts in gynaecological pathology (over 50% of working hours spent on gynecologic cases). All FS diagnoses were performed in accordance with guidelines proposed by Hart and Norris for mucinous tumors and by Scully for serous tumors [15, 16]. Definite diagnosis following frozen section was typically not performed by the same pathologist. Preoperative He4 serum value and ROMA score were assessed for patients referred to Padua University, excluding cases with known kidney impairment [17].

We excluded cases with prior history of neoplasia, adjuvant chemotherapy or radiation therapy, carriers of BRCA gene mutations, concomitant synchronous tubal, or other gynecological neoplasia. We also excluded all cases in which a second revision of paraffin-embedded tissue samples did not confirm the initial histological diagnosis.

The choice of surgical treatment depended on disease extension, patients' age, and desire of fertility preservation. Fertility sparing surgery implies that the uterus and at least part of one ovary were spared. Radical surgery comprises of total hysterectomy and bilateral salpingo-oophorectomy, infracolic omentectomy, peritoneal cytology, and biopsies with or without pelvic/lombo-aortic lymphadenectomy (in relation to the preoperative detection of bulky lymph nodes). In the event of mucinous histology, appendectomy was also performed.

Pathologists were informed of the results of preoperative clinical and instrumental assessment of all patients. For Group_A patients, additional information regarding He4 and CA 125 serum concentrations and ROMA score was available, whereas, for those in Group B, only the preoperative serum value of CA 125 was known.

The FS diagnosis was compared to the permanent pathology report. The FS diagnostic accuracy was defined as the number of cases with complete concordance between FS and final histology divided by the total number of cases. “Underdiagnosis” was defined by Song et al. as those cases diagnosed as benign or borderline on frozen section and subsequently corrected at permanent pathology as borderline or malignant, respectively. Contrarily, “overdiagnosis” was defined as a FS diagnosis of BOTs or ovarian malignancy later defined as benign and borderline, respectively.

### 2.1. Blood Sample Assays

Peripheral venous blood samples were obtained from all patients prior to surgery (within one week). Levels of both CA 125 and He4 were measured by fully automated ARCHITECT instrument (Abbott Diagnostics, ARCHITECT, Abbott Park, IL, USA). Cut-off values were set at 35 IU/L for CA 125 while for He4 at ≤70 pmol/L for pre-menopausal state and ≤140 pmol/L in menopausal state, according to the indications of the manufacturer. The ROMA cut-off values for high-risk patients were defined as >7.4% in premenopausal women and >25.3% in postmenopausal women [18].

### 2.2. Outcomes

The primary endpoint was to compare Group_A and Group_B in terms of FS diagnostic accuracy.

The secondary endpoint was to identify, in both study groups, the clinical-pathological (preoperative and intraoperative) factors associated with over- and underdiagnosis at frozen section.

### 2.3. Statistical Analysis

Statistical analysis was performed by SPSS (Chicago, IL) software for Windows version 19, using parametric and nonparametric tests, when appropriate. We performed the Kolmogorov-Smirnov to test normality of distribution. Continuous data were tested with the *t*-test, and categorical variables were tested with the *χ* ^2^ test or Fisher's exact test, when appropriate. The results obtained from the data collection were expressed in absolute numbers, percentages for discrete variables, and means ± standard deviations for continuous variables. We performed univariate logistic regression models to evaluate the effect of clinical-pathological parameters on frozen section analysis. We estimated a 95% CI by Wilson method using 2 × 2 tables. Statistical significance was defined as *P* < 0.05.

## 3. Results

In the time interval considered, a total of 119 cases were diagnosed with BOTs; of these, 113 satisfied the inclusion criteria and were enrolled into the study. 43 patients were assigned to Group_A, while the remaining comprised Group_B (39 from Parma University and 31 from Vicenza Hospital).

The mean age at diagnosis was 53.1 ± 16.0 years (ranging from 17 to 87 years) and BMI was 24.2 ± 3.8 (18–40). 19 patients were nulliparous (16.8%), 29 primiparous (25.7%), and 65 multiparous (57.5%); 59 patients (52.2%) were postmenopausal and 54 (47.8%) premenopausal. No differences were found between Group A and Group B regarding general features (Table 1).

Sonographic features of the pelvic masses were as follows: mean diameter was 11.9 ± 4.9 cm (ranging from 4 to 26 cm); 62 cysts were anechoic (54.9%) and 51 hypoechoic (45.1%); septa were found in 30 cases (26.5%), papillae were found in 29 cases (25.7%), and atypical vascularization was found in 30 cases (26.5%) (Table 2).

Preoperative serum CA125 mean value was 55.0 ± 76.0 UI/L (ranging from 1.6 to 474 UI/L) with 46% of the cases (52 patients) having a value greater than the cut-off (35 UI/L).

In Group_A patients, the mean preoperative He4 serum value was 89.9 ± 175.0 pmol/L (range: 21.6–1163.0) with 55.8% of the cases (24 patients) having a value greater than the cut-off adjusted for menopausal status. ROMA algorithm showed an increased risk for ovarian cancer in 30.2% of the cases (13 patients) (Table 3).

Laparoscopy was performed in 70.8% of the patients (80 cases of which 31/43 were in Group_A and 49/70 in Group_B) and laparotomy in 29.2% (33 cases). Primary surgery was cystectomy in 23% of the cases (26 patients of which 10/43 were in Group_A and 16/70 in Group_B) and salpingo-oophorectomy in 77% (87 patients of which 33/43 were in Group_A and 54/70 in Group_B).

The histological FS analysis detected 57 cases of serous BOTs (50.5%), 25 cases of mucinous BOTs (22.1%), and 1 case of carcinoma (0.9%) while 30 cases (26.5%) resulted negative for atypia.

The number of FS slides per specimen ranged between 3 and 5 for each, with a mean value of 4.14 in Group_A and 4.02 in Group_B. Postoperative definitive histology detected 62 cases of serous BOTs (54.9%), 37 cases of mucinous BOTs (32.7%), 7 cases of serous (6.2%), and 4 cases (3.5%) of mucinous carcinoma while 3 cases (2.7%) were negative for atypia. Peritoneal cytology was positive in 31% of cases (35 cases, 30 of which were in BOTs and 5 in cancer). The patients were classified according to FIGO staging system as stage IA in 63.6% (70 cases of which 64 were in BOTs and 6 in cancer), IB in 3.7% (4 cases all BOTs), and IC in 22.7% (25 cases of which 22 were in BOTs and 3 in cancer).

**Figure 1:**
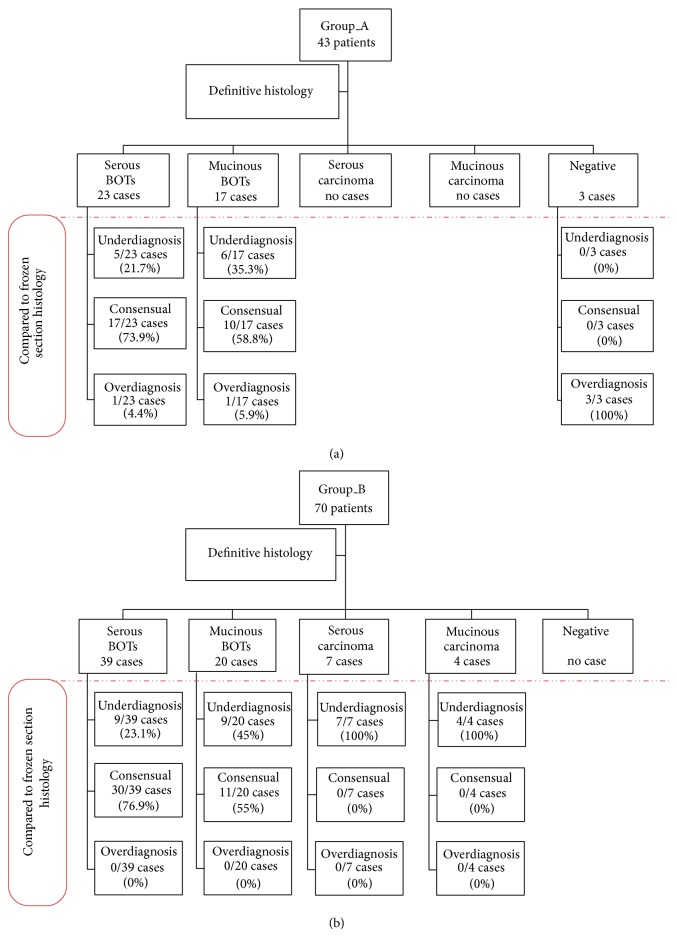
Flow diagram: comparison between diagnosis at frozen section and definitive histology stratified for histotype. (Group_A versus Group_B).

FIGO stage II-IV was assigned to 10% of cases (11 cases of which 9 were in BOTs 12 and 2 in cancer) (Figure 1).

Regarding the accuracy of FS compared to final histology, our study showed consensual diagnosis in 68 cases (60.2%), underdiagnosis in 41 cases (36.3%), and overdiagnosis in 4 cases (3.5%).

Comparison between the study groups in terms of FS diagnostic accuracy showed consensual diagnosis in 62.8% versus 58.6% of the cases, respectively (*P*: n.s.), underdiagnosis in 25.6% versus 41.4% (*P* < 0.05), and overdiagnosis in 11.6% versus 0% (*P* < 0.01). Detailed data are reported in Figure 1.

**Figure 2:**
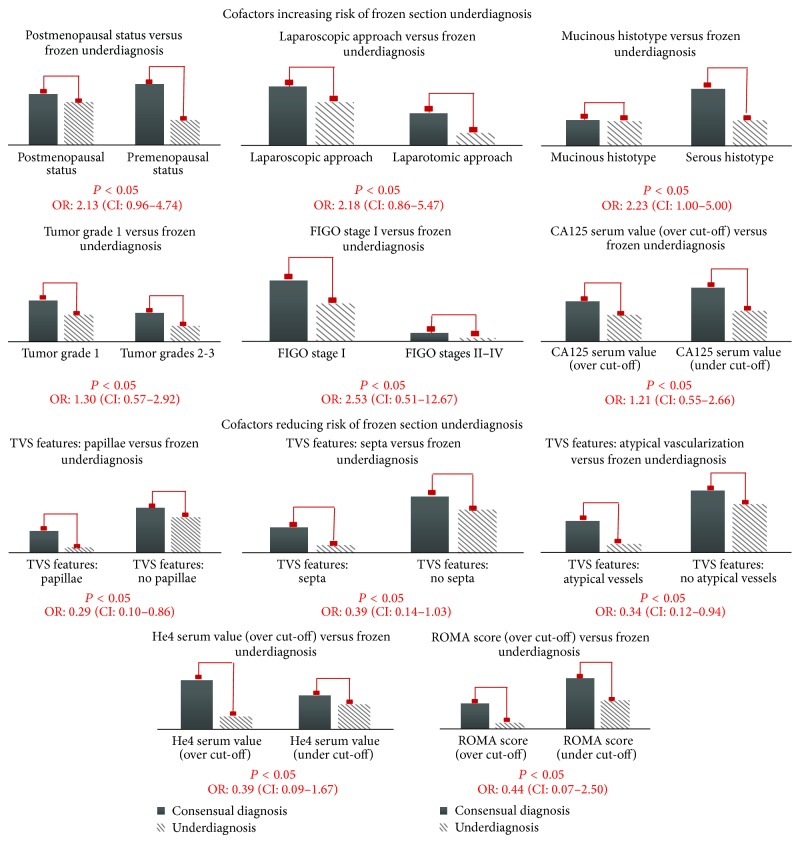
Cofactors increasing or reducing the risk of underdiagnosis at frozen section analysis.

Univariate analysis observed a statistically significant association between FS diagnostic accuracy and the following variables: menopausal status, ultrasound detection of papillae/septa/atypical vascularization, surgical approach, histotype, grading, and FIGO stage (*P* < 0.05). Considering underdiagnosis as the major risk factor for low FS accuracy, we calculated the ODDS ratio (OR) for every single clinical-pathological cofactor, excluding from the 2 × 2 table the 5 cases of overdiagnosis. Risk factors for underdiagnosis were: menopausal status (OR: 2.13; CI: 0.96–4.74), laparoscopic approach (OR: 2.18; CI: 0.86–5.47), mucinous histotype (OR: 2.23; CI: 1.00–5.00), low grading (OR: 1.30; CI: 0.57–2.92), and FIGO stage I (OR: 2.53; CI: 0.51–12.57). Protective factors for underdiagnosis were represented by ultrasound detection of papillae (OR: 0.29; CI: 0.10–0.86), septa (OR: 0.39; CI: 0.14–1.08), and atypical vascularization on color-Doppler (OR: 0.34; CI: 0.12–0.94). Concerning the association between preoperative serum values of CA125, He4, and ROMA score (the last two only for GROUP_A) and risk of underdiagnosis at FS analysis, we found an OR: 1.21 (CI: 0.55–2.66) for CA125, an OR: 0.39 (CI: 0.09–1.67) for He4, and an OR: 0.44 (CI: 0.07–2.50) for ROMA score (Figure 2).

## 4. Discussion

The appropriate surgical management for ovarian tumors requires an accurate histological diagnosis. Benign lesions are usually managed conservatively while the radicality of treatment chosen for malignant tumors depends on treatment intention (curative or palliative) and on tumoral response to neoadjuvant/adjuvant therapy. Management of BOTs is usually “personalized” taking into account menopausal status and future fertility desire. Since treatment options range from conservative cystectomy or unilateral salpingo-oophorectomy (eventually associated with resection of peritoneal deposits and omental biopsy) to demolitive total hysterectomy, bilateral salpingo-oophorectomy, and omentectomy with or without appendectomy (excluding cases in which patients require fertility sparing approach), histology remains the most important factor guiding the surgeon's choice of treatment [1, 19, 20].

Intraoperative frozen section histological evaluation of a pelvic mass is crucial in determining the appropriate surgical procedure. While frozen section has a high overall accuracy for ovarian neoplasms, it is less reliable in borderline tumors [21]. Recent studies reported that the sensitivity of FS for benign and malignant ovarian lesions varies from 65% to 97% and from 71% to 100%, respectively, and the specificity from 97% to 100% and from 98.3% to 100% for benign and malignant lesions, respectively. Unfortunately, FS accuracy in cases of BOTs was relatively low: 25% sensitivity and 50% positive predictive value [8, 10, 22, 23]. Certain clinical-pathological cofactors have been demonstrated to influence the accuracy of FS in the diagnosis of BOTs. Houck et al. reported, as significant predictors of misdiagnosis, a histology different from serous histotype, tumor size greater than 20 cm, and tumor confined to the ovaries [24]. Tempfer et al. reported that only tumor size is a significant predictor of FS underdiagnosis, while histotype, serum CA125 level, and tumor stage are not [9]. Brun et al. reported as significant predictive factors for FS misdiagnosis mucinous histology tumor size larger than 10 cm, a borderline component greater than 10%, and even the pathologist's experience [25]. Our data are in agreement with that stated above. We report concordance between FS and definitive histology in 60.2% of the cases (over 70% for serous and about 50% for mucinous histotypes). Frozen section accuracy in the event of mucinous BOTs was very low, often resulting in underdiagnosis. Our data confirms this finding since all cases of nonconsensual diagnosis (all cases of “underdiagnosis”) occurred in patients with BOTs of the mucinous histotype (86% of Group_A patients and 100% of Group_B patients having mucinous histotype).

Unfortunately, mucinous histotype is not preoperatively predictable and it is not the only feature influencing FS misdiagnosis.

Our data showed that premenopausal status, low grade (grade I), low stage (FIGO stage I), higher preoperative CA125 serum value, and laparoscopic approach increased the overall risk of underdiagnosis. It is universally accepted that the use of an endobag to retrieve the specimen is mandatory to reduce the risk of port-site metastasis [26]. This procedure may create a distortion of the macroscopic features of a pelvic mass and the consequent inability to perform an accurate macroscopic evaluation [27–30]. Preoperative information, such as sonographic features, plays a major role in allowing the pathologist to distinguish BOTs from negative or cancerous mass at microscopic evaluation. In particular, our data confirm that the detection of papillae/septa/atypical vascularization at TVS is of importance in significantly reducing “underdiagnosis.” Despite the fact that ultrasound is highly dependent upon the operator's skills, although in some cases pelvic mass description is not performed according to standardized criteria, TVS represents an accurate diagnostic tool in the preoperative assessment of malignant suspicious [31].

MRI and CT imaging techniques have certain advantages over TVS, such as higher soft tissue contrast and lower interoperator variability. However, radiation exposure, use of contrast agents, need of patient immobilization, and the high costs associated with the relatively low availability of these alternative imaging techniques make MRI a scarcely used tool in preoperative assessment of pelvic masses [32].

In the past years various diagnostic algorithms based on serum markers have been proposed as reproducible, low-cost tools able to distinguish malignant from benign disease [6, 17, 33]. CA 125 as a tumor marker has proven usefulness for the clinical management of patients with ovarian cancer in following clinical course, evaluating effectiveness of treatment, and detecting disease recurrence. CA125, however, has a relatively low specificity with increased levels in up to 41% of premenopausal women with benign masses. The recently discovered serum marker He4 appears superior to CA125 in differentiating benign from malignant ovarian masses both in pre- and postmenopausal women [14, 34, 35]; studies have shown increased values in 50% of ovarian cancer patients who do not express CA125 [36].

The exciting results regarding the usefulness of He4 in the preoperative assessment of patients at increased risk of malignancy lead the search for tissue expression and consequent serum detection in patients affected by BOTs.

Nassir et al. did not find a correlation between He4 tissue expression and BOT histology, suggesting that the previous findings in invasive ovarian cancer research are not applicable to BOTs [14]. Moreover, several clinical studies reported no difference in He4 serum levels between patients with borderline tumors and those with benign masses. As of today the role of He4 in the diagnosis of borderline conditions remains controversial. To our knowledge there is no data in literature that reports on the influence of He4 serum measurement on the diagnostic accuracy of FS in the event of a clinically suspicious pelvic mass later confirmed as borderline on final pathology.

According to our data He4 measurement and ROMA score did not improve the rate of concordant diagnosis when compared to CA125 alone (62.8% versus 58.6%, resp.).

However, the rate of underdiagnosis in Group_A seemed significantly improved by preoperative He4 and ROMA score assessment (25.6% versus 41.4%, resp.). All cases of underdiagnosis in Group_A were negative at the FS but were diagnosed as BOTs at the definitive histology. On the other hand 100% of the cases of underdiagnosis in Group_B were initially defined BOTs at FS and subsequently identified as carcinoma at definitive histology.

Despite the fact that our data require further evaluation on a large scale population, we suggest that He4 serum values and ROMA score should be part of the routine preoperative assessment of pelvic masses which present with or without characteristics suggestive of malignancy. Moreover, as “Achille's heel” of He4 and ROMA score performance, we found an increased risk of overdiagnosis on frozen section (11.6% versus 0%, resp.) despite the fact that 60% of these occurred in cases defined as borderline on FS and later negative at the definitive histology (all cases occurred in premenopausal women).

Our data, in agreement with FIGO guidelines, recommends caution in performing radical surgery when the diagnosis of BOTs is rendered by frozen section and doubts regarding accuracy may exist, particularly when histotype is mucinous and fertility is desired.

On the contrary, in postmenopausal patients frozen section may be safely employed as a diagnostic tool and, in cases of overdiagnosis, the ensuing surgical procedure does not result in “overtreatment.” In fact, in this cohort of patients the standard surgical staging is similar for both early stage ovarian cancer and BOTs.

The strengths of our study are represented by novelty of the aim, multicenter data collection, accuracy of preoperative and intraoperative available data, strict inclusion criteria, performance of FS diagnosis by dedicated pathologists, dosage of serum He4 samples in a single laboratory center, and homogeneity of the two groups in terms of general features.

As the limitations to our study we report FS and definitive histology performed by three different pathology units, small sample size, unbalanced number of patients comprising Group_A and Group_B, retrospective design, high rate of laparoscopic approach, absence of preoperative MRI investigation, exclusion of histotypes different from serous and mucinous, exclusion of cases presenting with bilateral pelvic masses, and lack of followup data in terms of recurrence and survival.

## 5. Conclusion

According to our data and pending further studies, we suggest He4 and ROMA score to be considered as routine preoperative assessment in the event that an ovarian mass is diagnosed and malignancy cannot be excluded. Information pertaining to serum marker assays and sonographic features may assist the pathologist in correctly diagnosing a pelvic mass on FS reducing the rate of underdiagnosis but potentially increasing the rate of overdiagnosis. The overall accuracy rate of FS in diagnosing BOTs (especially when the reported risk factors are found) remains low. Fertility sparing surgery is strongly suggested in patients with future desire of fertility. In postmenopausal women surgical staging for BOTs is acceptable even in cases of overdiagnosis at FS since surgical treatment is similar for both early stage ovarian cancer and BOTs.

## Figures and Tables

**Table 1:** General features: comparison between the groups (Group_A versus Group_B).

		Number	Mean value	Standard deviation	*P* value
Age	Group_A	43	52.91	16.72	n.s
	Group_B	70	53.27	15.75	

BMI	Group_A	43	24.42	3.51	n.s
	Group_B	70	24.09	3.99	

			Category	Number (%)	*P* value

Parity	Group_A	43	Nulliparous	8 (18.6)	n.s
			Primiparous	6 (14.0)	
			Multiparous	29 (67.4)	
	Group_B	70	Nulliparous	11 (15.7)	
			Primiparous	23 (32.9)	
			Multiparous	36 (51.4)	

Hormonal status	Group_A	43	Premenopausal	20 (46.5)	n.s
			Postmenopausal	23 (53.5)	
	Group_B	70	Premenopausal	39 (55.7)	
			Postmenopausal	31 (44.3)	

**Table 2:** Preoperative ultrasound features of the pelvic mass: comparison between the groups (Group_A versus Group_B).

TVS morphological features		Number	Mean value	Standard deviation	*P* value
Size of pelvic mass (TVS investigation)	Group_A	43	12.33	4.48	n.s
	Group_B	70	11.77	5.18	

TVS morphological features			Category	Number (%)	*P* value

Echogenicity	Group_A	43	Anechoic	24 (55.8)	n.s
			Hypoechoic	19 (44.2)	
	Group_B	70	Anechoic	38 (54.3)	
			Hypoechoic	32 (45.7)	

Intracystic septa	Group_A	43	Yes	23 (32.9)	n.s
			No	47 (67.1)	
	Group_B	70	Yes	7 (16.3)	
			No	36 (83.7)	

Intracystic papillae	Group_A	43	Yes	22 (31.4)	n.s
			No	48 (68.6)	
	Group_B	70	Yes	7 (16.3)	
			No	36 (83.7)	

Atypical vascularization	Group_A	43	Yes	23 (32.9)	n.s
			No	47 (67.1)	
	Group_B	70	Yes	7 (16.3)	
			No	36 (83.7)	

**Table 3:** Preoperative serum value of CA125 and He4 biomarkers: absolute value and risk estimation according to predefined cut-off (in relation to hormonal status for He4) (only for CA125: Group_A versus Group_B).

		Number	Mean value	Standard deviation	*P* value
Preoperative CA125 serum value (UI/L)	Group_A	43	52.92	83.26	n.s
	Group_B	70	56.32	71.85	

Preoperative He4 serum value (pmol/L)	Group_A	43	89.99	175.07	—
	Group_B	—	—	—	

			Category	Number (%)	*P* value

CA125 cut-off value	Group_A	43	Over	15 (34.9)	n.s
			Under	28 (65.1)	
	Group_B	70	Over	37 (52.9)	
			Under	33 (47.1)	

He4 cut-off value	Group_A	43	Over	24 (55.8)	—
			Under	19 (44.2)	
	—	—	—	—	
			—	—	

ROMA score cut-off value	Group_A	43	Over	13 (30.2)	—
			Under	30 (69.8)	
	—	—	—	—	
			—	—	
